# Case Report: Miles Surgery Ameliorates High Blood Pressure in a Rectal Carcinoma Patient With Essential Hypertension

**DOI:** 10.3389/fcvm.2021.762959

**Published:** 2021-11-02

**Authors:** Meng-Wan Zhang, Bo-Shi Fan, Jian-Guang Yu

**Affiliations:** ^1^Department of Pharmacy, Shanghai Chest Hospital, Shanghai Jiao Tong University, Shanghai, China; ^2^Department of Thoracic Surgery, Sixth Medical Center of PLA General Hospital, Beijing, China

**Keywords:** hypertension, miles surgery, sympathetic tone, gut microbiota-brain axis, case report

## Abstract

Hypertension is one of the major causes of public health problems. Multiple factors affecting gastrointestinal tract function are involved in hypertension. Emerging studies have manifested that gut intervention may play significant roles in regulating blood pressure but the underlying mechanisms are complex and not fully clear. Here, we report a case of 66 years old male who had a long history of hypertension and received Miles surgery for rectal carcinoma. The blood pressure of this patient was returned to normal levels after the operation. The possible reasons could be the modulation of sympathetic tone and the gut microbiota-brain axis. This report provides evidence about the relevance between hypertension and gut intervention particularly in the colorectal sites and gives hints for investigating the possible mechanisms of hypertension and the novel strategy for blood pressure control.

## Introduction

Hypertension, a major health problem all over the world, causes a variety of cardiovascular diseases and metabolic diseases, and the prevalence rate is rising (1–3). However, the reason for essential hypertension remains unclear. Mounting researches argue that the external and internal factors remodulate the central nervous system and result in elevation of the sympathetic tone. The elevated sympathetic tone initiates and maintains the pathogenesis and development of hypertension (4). Roux-en-Y gastric bypass (RYGB) surgery, widely applied to treat refractory obesity, is reported to attenuate high blood pressure (HBP) in hypertensive patients with obesity and spontaneously hypertensive rats (SHRs) through antagonizing the renal sympathetic nervous activity (RSNA) (5). We report that a patient with a 40-year history of hypertension was diagnosed with rectal carcinoma and colon multiple polyps. The blood pressure decreased to a normal level after the Miles surgery, a complicated surgery of abdominoperineal resection for carcinoma of the rectum and the terminal portion of pelvic colon (6, 7).

## Case Description

The male patient was 66 years old. He suffered from hypertension for 40 years and the blood pressure was up to 155/105 mmHg when untreated. He had been taking 75 mg captopril and 3.6 g salvia tablet daily to maintain the blood pressure of about 130/75 mmHg in daily life. The body mass index (BMI) was 21.5 kg/m^2^ before the operation. He was diagnosed with rectal carcinoma and colon multiple polyps. The main diagnostic methods are computed tomography and electronic colonoscopy examination. Computed tomography demonstrated a tumor in the colon. Electronic colonoscopy examination revealed a 0.5 × 0.5 cm polyp on transverse colon and sigmoid separately. A nodular bulge could be seen, 4 cm from the anus at 6 o'clock position when the patient was on left lateral position, and the surface eroded. Hemorrhoids and anal fissure were excluded through the differential diagnoses. The patient received Miles surgery for removal of rectum and distal sigmoid colon. The histopathological results displayed adenocarcinoma. The blood pressure was 125/75 mmHg the next day after the operation, with no anti-hypertensive therapy, and the BMI was 20.5 kg/m^2^. The patient recovered smoothly and was discharged 10 days postoperatively and began to receive the postoperative chemotherapy program with oxaliplatin, 5-fluorouracil and calcium folinate 3 weeks after the operation. In one course, oxaliplatin (85 mg/m^2^) and calcium folinate (400 mg/m^2^) was taken on the first day, while 5-fluorouracil was taken for 3 days (400 mg/m^2^ on the first day and 1,200 mg/m^2^ for the next 2 days), repeating every 2 weeks. The patient took 6 courses of postoperative chemotherapy totally for 3 months. The patient tolerated the postoperative chemotherapy during the 3 months courses with mild adverse reactions such as nausea and vomiting but without severe adverse reactions such as serious renal or hepatic impairments. A several-month telephone follow-up was conducted after he was discharged and no recurrence and metastasis of the rectal carcinoma was found based on computed tomography, electronic colonoscopy examination, and tumor markers tests including normal levels of carbohydrate antigen 199, carbohydrate antigen 125, carbohydrate antigen 153, carcinoembryonic antigen, and α-fetoprotein through the whole course.

**Figure 1:**
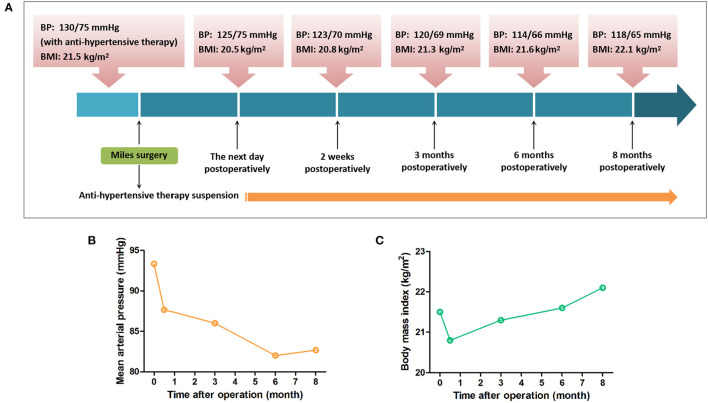
Timeline for blood pressure and BMI of the patient. **(A)** The blood pressure and BMI of the patient during the follow-up are showcased as a timeline. **(B)** The blood pressure of the patient indicated as the mean arterial pressure had been maintained normal without anti-hypertensive drug treatment postoperatively. **(C)** BMI of the patient decreased lower than preoperative level 2 weeks postoperatively and then recovered 3 months after the operation.

More attention was paid to the recurrence and metastasis of the rectal carcinoma at the beginning. Subsequently, we found the blood pressure of the patient had been maintained in the normal range without anti-hypertensive drug treatment. Blood pressure was measured at 3–5 pm and the BMI was calculated according to the body weights, recorded respectively at 2 weeks, 3, 6, and 8 months after the operation. Blood pressure of the patient decreased to normal level since 2 weeks postoperatively and has been normal until now without any anti-hypertensive treatment. The relevant data collected during the hospitalization and follow-up have been showcased as a timeline in Figure 1, as well as the variation trends of the blood pressure and BMI of the patient during the follow-up. Family history revealed no history of cardiac or other congenital abnormalities. Past history recorded no alcoholic intake or smoking.

## Discussion

We report that Miles surgery can ameliorate the HBP of the patient. Since the patient has a 40-year hypertension history, rectal carcinoma might not be the cause of his hypertension. After the cessation of the chemotherapy, the blood pressure varies between 110–135/55–80 mmHg, demonstrating that chemotherapy is likely not the reason for the amelioration of HBP. The reason for the reduced blood pressure the next day postoperatively might be the influence of operation such as post-operative bleeding and anesthesia impacts. The BMI of the patient was lower than preoperative level at 2 weeks after the operation but recovered to a stable level in the following months, indicating that the drop on blood pressure could not be due to BMI alteration. Recently, hypertension is proved to be related to impaired metabolic homeostasis and dysfunction of the gut such as gut autonomic nerve activity, gut microbiota alteration, and gut hormones secretion. Intervention in the gastrointestinal tract could ameliorate hypertension and related cardiometabolic diseases (8–10). Previous researches on the relationship between gut intervention and blood pressure focused on metabolic surgery, such as sleeve gastrectomy (SG) and RYGB surgery (11, 12). The bypass surgery is probable to lower blood pressure through regulating gut hormones secretion and gut microbiota, as well as reducing sympathetic nervous system activity (5, 13–15).

A variety of factors, including dietary elements, gut microbiota, and hormones, exert an impact on the nervous system through the gut-brain cross-talk. Primary afferent neurons, immune cells, and enteroendocrine cells in the gut send the information to the central nervous system. The brain communicates to the viscera, including the gastrointestinal tract, through the autonomic nervous system, hypothalamic-pituitary-adrenal axis, and sympatho–adrenal axis (16, 17). Elevation of sympathetic tone accounts for the pathogenesis and maintenance of hypertension (18). Studies indicate that RYGB surgery transmits a signal to the nucleus of solitary tract (NTS). Remodulation of NTS neurons reduces RSNA, baroreflex, and other factor-induced changes in heart rate, blood pressure, and vasoconstriction, as well as ameliorates cardiac remodeling and dysfunction, which lowers blood pressure in the SHRs (5). Glucagon-like peptide 1 (GLP-1) is secreted by the ileum cells and markedly increases at 3 months after SG and RYGB surgery (19). GLP-1 receptor agonists, exenatide and liraglutide, are proved to lower blood pressure. Exenatide prevents acute angiotensin II-induced extracellular regulated protein kinase phosphorylation in proximal tubular cells of kidney, and angiotensin II plays the key role in the over-activity in the sympathetic nervous system. However, the mechanism that metabolic surgery promotes GLP-1 secretion remains unclear. Moreover, exenatide and liraglutide have been reported to reduce body weight, and thus decrease blood pressure (20). However, the BMI of our patient varied between 20.8 and 21.5, and after regain of the weight, the blood pressure was still normal, indicating that BMI change has no effect on the amelioration of the HBP.

**Figure 2:**
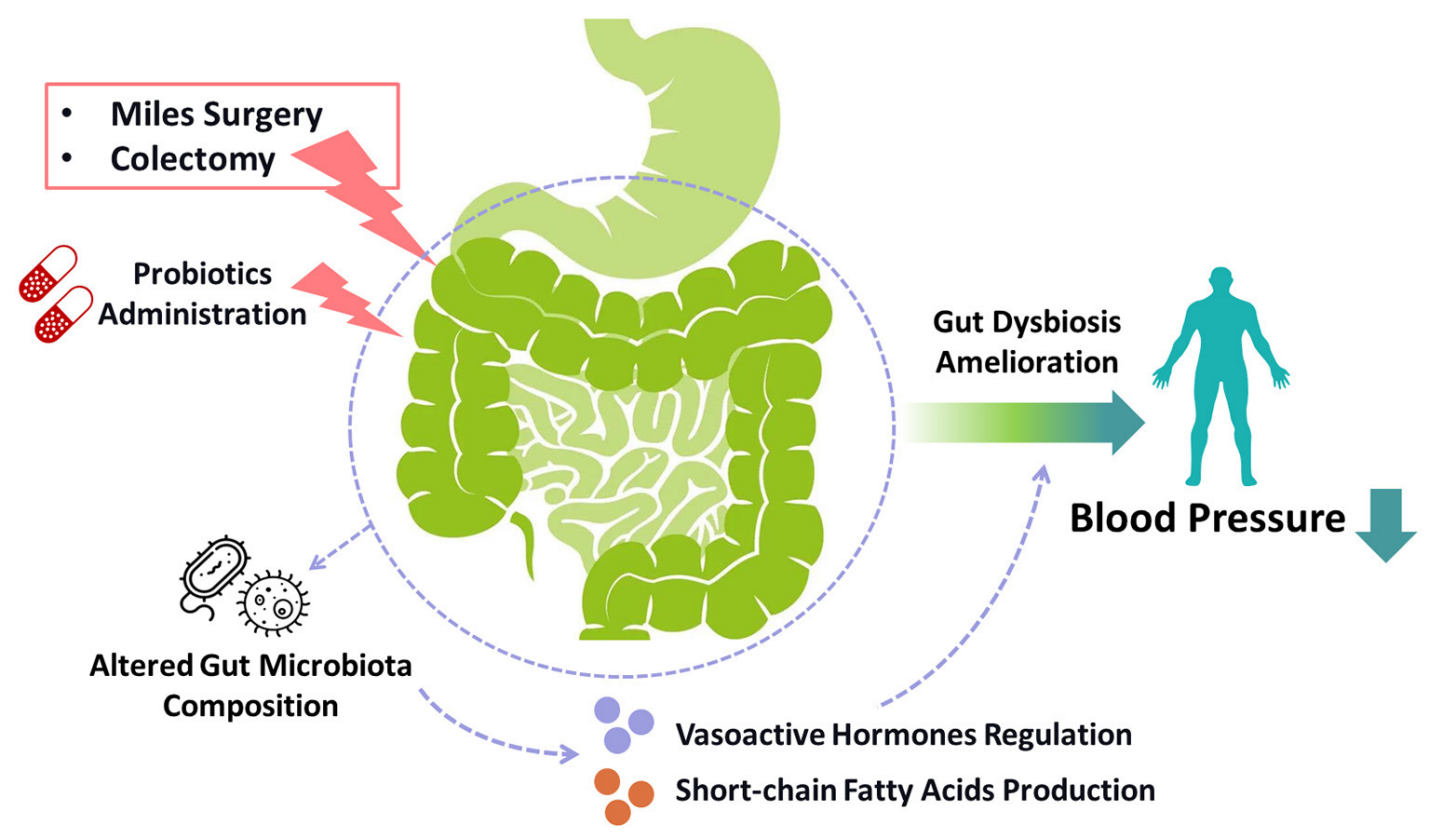
Gut intervention-induced microbiota alteration in blood pressure control. Gut interventions targeting colorectal regions such as miles surgery and colectomy, as well as probiotics administration, may ameliorate hypertension by modulating gut dysbiosis. These gut surgeries and exogenous probiotic supplements altered gut microbiota composition to regulate the production of vasoactive hormones and short-chain fatty acids, which could be the underlying mechanisms of the reduction of blood pressure. The administration of probiotics might also be a novel strategy for treating hypertension.

A mounting number of researches on human and rats demonstrate that the quantity, types, and DNA variety of gut microbiota significantly affect blood pressure. Gut dysbiosis has been found in hypertensive human and animals (21–23). Recent investigations suggest that gut microbiota could affect blood pressure through producing vasoactive hormones and short-chain fatty acids which bind to the GTP-binding protein-coupled receptors to regulate blood pressure (24–27). Furthermore, the administration of probiotics in human may help to decrease blood pressure, probably by generating short-chain fatty acids and γ-aminobutyric acid, and thus gut microbiota modulation could be a promising strategy for treating hypertension (28, 29). Gastrointestinal surgeries such as RYGB surgery could have an influence on gut microbiota composition (26). A cohort study demonstrates that colectomy could reduce the risk of hypertensive disorders, hinting the possible relevance between gut microbiota and hypertension (30). Therefore, these improve the possibility that Miles surgery may change the microbiota-brain axis and thus decrease blood pressure (Figure 2).

In summary, we report that Miles surgery can ameliorate the HBP of the patient with rectal carcinoma. Inhibition of the sympathetic tone and change of the gut microbiota-brain axis may account for the phenomenon. However, more cases and experiments are needed to confirm the phenomenon including whether and how Miles surgery could influence the gut microbiota or circulating metabolites, as well as the sympathetic nerve activity. We will also try to explore the underlying molecular mechanisms such as investigating the specific modulative manners of gut microbiota on blood pressure and which nerves or neurotransmitters in sympathetic nerve system are involved in the regulation of blood pressure by Miles surgery in the future.

## Data Availability Statement

The original contributions presented in the study are included in the article/supplementary material, further inquiries can be directed to the corresponding authors.

## Ethics Statement

Ethical review and approval was not required for the study on human participants in accordance with the local legislation and institutional requirements. The patients/participants provided their written informed consent to participate in this study.

## Author Contributions

M-WZ: conceptualization and writing—original draft. B-SF: data collection. J-GY: conceptualization and writing—review and editing. All authors contributed to the article and approved the submitted version.

## Funding

This work was supported by grants from the Nurture projects for basic research of Shanghai Chest Hospital (2020YNJCQ01 and 2020YNJCM08) and the National Natural Science Foundation of China (82173812 and 82104161).

## Conflict of Interest

The authors declare that the research was conducted in the absence of any commercial or financial relationships that could be construed as a potential conflict of interest.

## Publisher's Note

All claims expressed in this article are solely those of the authors and do not necessarily represent those of their affiliated organizations, or those of the publisher, the editors and the reviewers. Any product that may be evaluated in this article, or claim that may be made by its manufacturer, is not guaranteed or endorsed by the publisher.
